# Fisetin Prevents Acetaminophen-Induced Liver Injury by Promoting Autophagy

**DOI:** 10.3389/fphar.2020.00162

**Published:** 2020-02-28

**Authors:** Jiaqi Zhang, Licong Zhao, Cheng Hu, Tao Wang, Juan Lu, Chenqu Wu, Long Chen, Mingming Jin, Hao Hu, Guang Ji, Qin Cao, Yuanye Jiang

**Affiliations:** ^1^ Department of Gastroenterology, Putuo Hospital, Shanghai University of Traditional Chinese Medicine, Shanghai, China; ^2^ Department of Second Clinical College, China Medical University, Shenyang, Liaoning, China; ^3^ Experiment Center for Science and Technology, Shanghai University of Traditional Chinese Medicine, Shanghai, China; ^4^ Shanghai University of Medicine & Health Sciences of Integrated Traditional Chinese and Western Medicine, Shanghai University of Traditional Chinese Medicine, Shanghai, China; ^5^ Department of Plastic and Reconstructive Surgery, East Hospital, Tongji University, Shanghai, China; ^6^ Institute of Digestive Diseases, Longhua Hospital, Shanghai University of Traditional Chinese Medicine, Shanghai, China

**Keywords:** acetaminophen, liver injury, fisetin, autophagy, ATG5

## Abstract

Acetaminophen (APAP) overdose is a leading cause of drug-induced acute liver failure in clinical and hospital settings. Fisetin (FST) is a phenolic compound derived from natural products such as fruit and vegetables. Our research investigated the protective mechanisms of FST in APAP-induced hepatic injury *in vitro* and *vivo*. Assessment of mouse serum levels of alanine/aspartate aminotransferases (ALT/AST), liver myeloperoxidase (MPO) activity, malondialdehyde (MDA), glutathione (GSH), and reactive oxygen species (ROS) demonstrated the protective effects of FST toward APAP-induced liver injury. FST also reversed an APAP-induced decrease in mouse L-02 cell line viability. Our results also showed that FST significantly promoted APAP-induced autophagy and inhibited inflammasome activation both *in vivo* and *in vitro*. We also found that silencing ATG5, using si-ATG5, reduced the protective effects of FST against APAP-induced hepatotoxicity and reversed the effects on autophagy. Finally, we used the autophagy inhibitor, 3-methyladenine (3-MA) to validate the involvement of autophagy in FST against APAP-induced hepatotoxicity *in vitro*. We demonstrated that FST prevented APAP-induced hepatotoxicity by increasing ATG5 expression, thereby promoting autophagy and inhibiting inflammasome activation.

## Introduction

Acetaminophen is an antipyretic analgesics ingredient, which is safe to use when correctly adhering to prescribed therapeutic dosages. However, acetaminophen (APAP) overdosing can cause serious hepatic injury in animals and humans (Larson et al., 2005; Blieden et al., 2014; Jaeschke et al., 2014). APAP comes in a variety of formulations as well as prescription and over-the-counter combinations. Its wide availability makes patients more likely to consume more than the recommended dose of the drug. Approximately half of patients with APAP liver toxicity experience mild to moderate reactions, while approximately 48% of patients are diagnosed with acute liver injury. Hence, APAP has become one of the most widely reason of acute liver injury in the United States and other western countries (Bernal, 2003; Fontana, 2008; Canbay et al., 2009; Blieden et al., 2014). APAP forms a highly reactive metabolite, N-acetyl-p-benzoquinoneimine (NAPQI), which induces hepatotoxicity (Alonso et al., 2015). Although cellular reduced glutathione (GSH) has been shown to detoxify this metabolite, it becomes depleted once APAP is overdosed and forms excessive NAPQI. Superabundant NAPQI binds with free thiol groups in proteins, leading to reactive oxygen species (ROS) accumulation and subsequent disturbances in cellular redox homeostasis (Cohen et al., 1997; Gunawan and Kaplowitz, 2007; Jaeschke et al., 2012).

Inflammasomes are intracellular multi-protein complexes which regulate the activation of caspase-1 and in turn, promote the maturation and secretion of the cytokine precursors, pro-interleukin (IL)-1 and pro-IL-18 during natural immune defenses (Martinon et al., 2002). It has been shown that after an APAP overdose, both mice and humans undergo inflammatory responses caused by inflammasomes (Woolbright and Jaeschke, 2017). However, if these inflammasomes are inhibited, APAP-induced hepatocyte death may be prevented (Kim and Park, 2018).

Autophagy plays key roles in survival mechanisms for adverse intracellular events, like nutriment or growth factor deprivation. The autophagy effect selectively removes damaged organelles, especially mitochondria, therefore autophagy protects against cell damage caused by mitochondria-induced cell death (Lum et al., 2005; Tan et al., 2018). APAP overdose causes liver injury by inducing mitochondrial damage and associated necrosis in liver cells. Former studies have shown that high does APAP leads to the activation of autophagy. Interestingly, further promoting autophagy alleviated hepatic cell injury caused by APAP *via* eliminating injured mitochondria (Ni et al., 2012; Lin et al., 2014; Yan et al., 2018). When autophagy is suppressed, APAP mediated liver damage can be aggravated, however rapamycin-promoted autophagy has been found to mitigate APAP induced liver toxicity (Ni et al., 2012).

**Figure 1:**
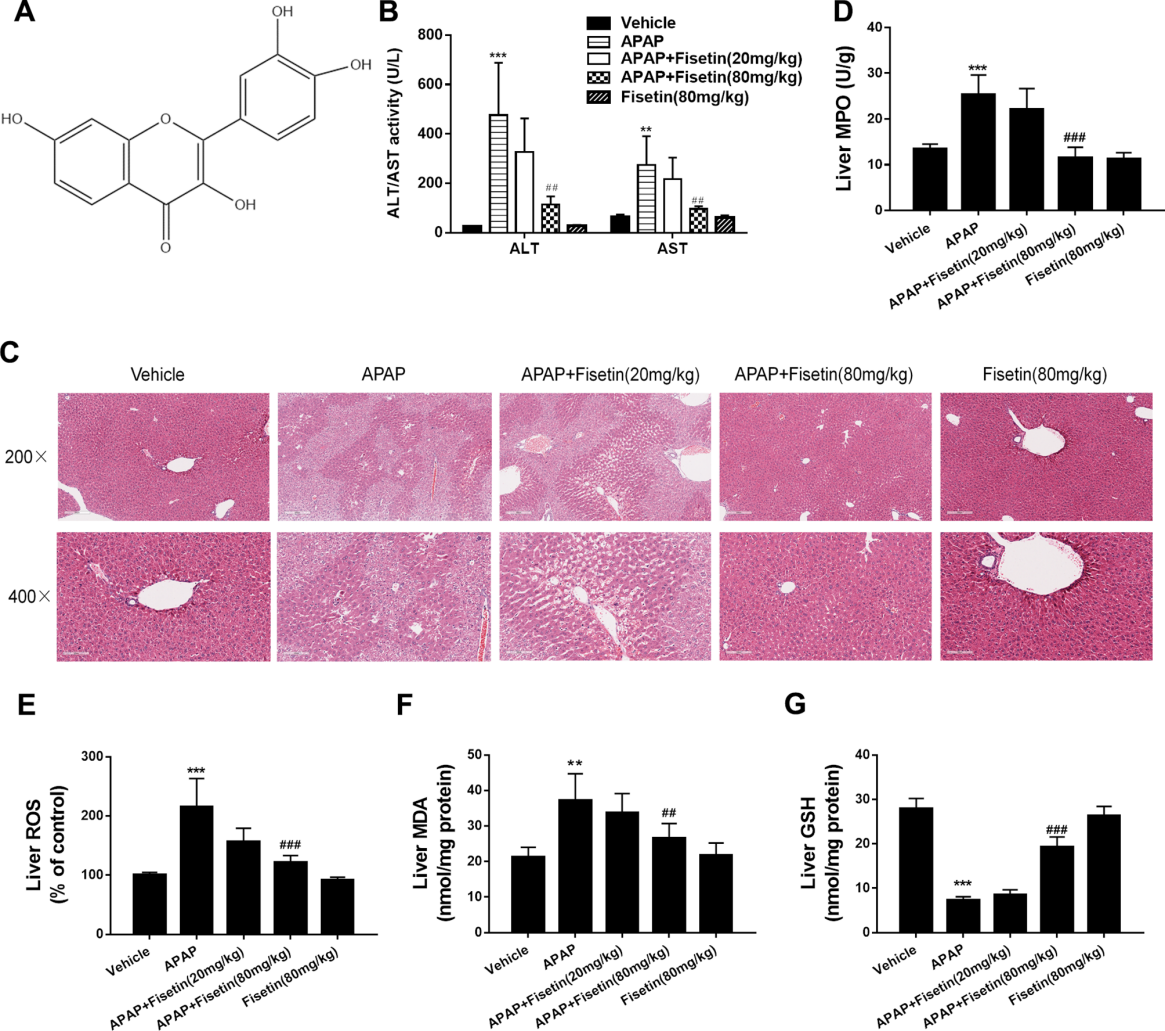
Fisetin (FST) prevents acetaminophen (APAP)-induced liver injury in mice. **(A)** The structure of FST. The molecular formula of FST is C_15_H_10_O_6_ and the molecular weight is 286.23 g/mol. **(B)** Serum alanine/aspartate aminotransferases (ALT/AST) levels at 6 h after APAP administration. **(C)** Representative images of H&E stained liver sections (×200 and ×400 magnification). **(D)** Liver myeloperoxidase (MPO) activity, **(E)** liver reactive oxygen species (ROS) levels, **(F)** liver malondialdehyde (MDA) activity, and **(G)** liver glutathione (GSH) levels. Data are expressed as mean ± SEM (n = 8). ^**^P < 0.01, ^***^P < 0.001 compared to vehicle; ^##^P < 0.01, ^###^ P< 0.001 compared to APAP.

Fisetin (FST) is a flavonoid polyphenol. It was found in some vegetables and fruits such as lettuce, cucumbers, strawberry, and grapes (Sak et al., 2016). Previous studies have shown that FST has anti-tumor (Ferreira de Oliveira et al., 2018; Sun et al., 2018), anti-oxidation (Singh et al., 2018; Zhang et al., 2018), anti-inflammatory (Wu et al., 2017; Huang et al., 2018), and other pharmacological effects. It has been reported that FST inhibits inflammation related cytokines like TNF-α, IL-6, and NF-κB in streptozotocin (STZ)-induced diabetic cardiomyopathy rats (Althunibat et al., 2019). FST has also been shown to upregulate glutathione (GSH) in isoproterenol-induced cardiac ischemic injury rats and hyperhomocysteinemia-induced endothelial dysfunction rats (Hemanth Kumar et al., 2017; Garg et al., 2019). Thanks to its chemical structure (**Figure 1A**), FST has an electron donating capacity to scavenge free radicals, which could have implications for diseases caused by oxidative stress (Khan et al., 2013). In a previous study, we showed that FST alleviates hepatic lipid metabolism through activation of the SIRT1 pathways (Liou et al., 2018). Similarly, FST has been observed to induce autophagy in multi cancers like breast cancer, oral cell carcinoma, and pancreatic cancer (Yang et al., 2012; Kim et al., 2016; Klimaszewska-Wisniewska et al., 2016; Jia et al., 2019; Park et al., 2019). However, the autophagy effects of FST on liver injury are still unclear.

Hence, we demonstrated protection mechanisms of FST against acute hepatic injury caused by acetaminophen.

## Materials and Methods

### Reagents and Chemicals

FST of 98% purity was purchased from Shanghai Hitsanns Co., Ltd (Shanghai, China). Kits for the analysis of alanine/aspartate aminotransferases (ALT/AST), malondialdehyde (MDA), myeloperoxidase (MPO), and GSH were obtained from Nanjing Jiancheng (Nanjing, China). H2DCFDA, RPMI1640, and fetal bovine serum (FBS) were obtained from Life Technology (Carlsbad, CA, USA). The Pierce^®^ BCA Protein Assay Kit was purchased from Thermo Fisher Scientific (Waltham, MA, USA). Whole cell protein extraction kits and enhanced chemiluminescence kits were obtained from Millipore (Darmstadt, Germany). Antibodies for immunoblotting including β-actin (#4970), LC3 (#2775), p62 (#88588), ATG5 (#2630), caspase-1 (#3866), IL-1β (#12242) were purchased from Cell Signaling Technology (Danvers, MA, USA) (all 1:1,000 dilutions). Enzyme-linked immunosorbent assay (ELISA) kits were purchased from RapidBio (West Hills, CA, USA). TRIzol reagent was purchased from Life Technology (Carlsbad, CA, USA). PrimeScript^®^ RT Master Mix and SYBR^®^ Premix Ex Taq^™^ were purchased from Takara (Shiga, Japan). APAP, NAPQI, MTT, and other reagents were obtained from Sigma-Aldrich (St. Louis, MO, USA) unless otherwise indicated.

### Animals

C57BL/6 mice (20 g ± 2) were purchased from the Shanghai Laboratory Animal Center of Chinese Academy of Sciences (Shanghai, China). Experimental animals were fed with a standard laboratory diet and given free access to tap water. They lived in a controlled room temperature (22°C ± 1), humidity (65% ± 5) with a 12:12 h light/dark cycle. All animals received humane care in compliance with institutional animal care guidelines approved by the Experimental Animal Ethical Committee of Shanghai University of Traditional Chinese Medicine. The protocol was reviewed and approved by the Experimental Animal Ethical Committee of Shanghai University of Traditional Chinese Medicine (Permit Number: PZSHUTCM190315014). All protocol was completed under sodium pentobarbital anesthesia, and all efforts were made to minimize animal suffering.

### Animal Treatment

Forty mice were randomly divided into five groups: 1) vehicle control, 2) APAP (400 mg/kg) (dissolved in saline), 3) APAP (400 mg/kg) + FST (20 mg/kg) (dissolved in CMC-Na), 4) APAP (400 mg/kg) + FST (80 mg/kg), and 5) FST (80 mg/kg). Mice were pre-administered orally with FST (20, 80 mg/kg per day) for seven consecutive days. On the last day, mice were orally administered a single dose of APAP (400 mg/kg) after administration of FST for 1 h. Animals were killed 6 h after APAP intoxication and plasma and liver tissues were collected.

### Blood and Tissue Collection and Biochemical Analysis

Anaesthetized mice were euthanized by cardiac puncture and blood was withdrawn. Immediately after cardiac puncture, livers were harvested. A portion of fresh liver tissue was fixed in 10% buffered formalin and the remaining tissue was snap frozen in liquid nitrogen and stored at −80°C. Blood samples were kept at room temperature for 2 h. Serum was collected after centrifugation at 840 g for 15 min. Serum ALT and AST were measured with kits according to manufacturer’s protocols. Liver MDA, MPO, ROS, and GSH were analyzed using commercial kits, according to manufacturers’ protocols.

### Liver Histological Observation

Mouse liver tissue samples were fixed in 10% phosphate buffered saline (PBS)-formalin for at least 24 h and embedded in paraffin for histological assessment. Samples were sectioned (5 μm), stained with hematoxylin and eosin (H&E), or Sirius Red using standard protocols. They were then examined microscopically for structural changes and observed under a light microscope (Olympus, Tokyo, Japan) to evaluate liver damage.

### Cell Culture

The L-02 cell line (passage number: 8) is derived from an adult human normal liver (Cell Bank, Type Culture Collection of Chinese Academy of Sciences, Shanghai). The human hepatocellular carcinoma cell line HepaRG (Wu et al., 2016) was purchased from Merck Millipore (Burlington, MA, USA). Both L-02 and HepaRG cell lines were cultured in RPMI1640 supplemented with 10% (v/v) fetal bovine serum, 2 mM glutamine, 100 U/ml penicillin, and 100 mg/ml streptomycin and incubated at 37°C in a humidified atmosphere containing 5% CO_2_.

### Cell Viability Assay

L-02 cells were plated into 96-well plates at an initial density of 5,000 cells per well and HepaRG cells were plated at an initial density of 3,000 cells per well. After attachment, cells were treated with FST dissolved in DMOS for 15 min and incubated with APAP or NAPQI for different times. After treatment, cells were incubated with 500 μg/ml MTT for 4 h. Blue formazan was dissolved in 10% sodium dodecyl sulfate (SDS), 5% isobutanol, and 0.01 M HCl, and plates were scanned on a microplate reader (Thermo Scientific) at 570 nm, with 630 nm as a reference wavelength. Cell viability was normalized as a percentage of control wells.

### Cell Transfection

To assess ATG5 expression, an ATG5 knockdown or corresponding negative control (si-NC) was constructed by GenePharma. L-02 cells were transfected with the si-ATG5 vector at a final concentration of 50 nM, using Lipofectamine 2000 (Invitrogen, Waltham, MA, USA) reagent according to manufacturer’s protocols.

### RNA Isolation and Quantitative Real-Time PCR

RNA was isolated using Tirol reagent (Invitrogen) according to manufacturer’s instructions and reversibly transcribed using the miScript Reverse Transcription kit (Qiagen). QRT-PCR was performed using a SYBR Premium Ex Taq II kit (Takara, Dalian, China) on an ABI PRISM 7500 Sequence Detection System (Applied Biosystems). All reactions were performed in triplicate and the mean value was used to calculate expression levels after normalization to β-actin.

### Protein Extraction and Western Blot Analysis

L-02 cells were lysed using radioimmunoprecipitation assay (RIPA) buffer and protein concentration was determined using the BCA protein assay kit. Approximately 30 μg of protein from each sample was separated using a 10% SDS-polyacrylamide gel and transferred to polyvinylidene fluoride (PVDF) membranes. Membranes were blocked with 5% skimmed milk in Tris buffered saline buffer with Tween 20 (TBST) and incubated with primary antibodies overnight at 4°C. Membranes were incubated with the corresponding secondary antibody for 1 h at room temperature and washed in TBST. Protein signals were detected using the Super ECL Plus Detection Reagent.

### Enzyme-Linked Immunosorbent Assay (ELISA)

Concentration of IL-1β and IL-18 in blood were measured by ELISA kit (Nanjing Jiancheng Bioengineering Institute) in consonance with standard protocols.

### Autophagic Flux Analysis

L-02 cells transfected with mRFP-GFP-LC3 were fixed with 4% paraformaldehyde and stained with 10 μM Hoechst 33342. Cell images were obtained using the Operetta High Content Imaging System (Perkin-Elmer) and analyzed using Harmony Analysis Software (Perkin-Elmer). Cells were analyzed using green (GFP) or red (mRFP) fluorescence. Autophagosomes were stained yellow puncta and autolysosomes stained red puncta in merged images. Autophagic flux was determined by the increased percentage of red puncta in merged images.

### Electron Microscopy

Cells were fixed with 2.5% glutaraldehyde in phosphate buffer and stored at 4°C, until embedding. Cells were post-fixed with 1% osmium tetroxide followed by an increasing gradient dehydration step using ethanol and acetone. Cells were then embedded in Araldite, ultrathin sections were obtained (50–60 nm), placed on uncoated copper grids and stained with 3% lead citrate-uranyl acetate. Images were examined using a CM-120 electron microscope (Philips).

### Flow Cytometry

To detect inflammasomes in hepatocytes, caspase-1 activity was determined using the FAM-FLICA *in vitro* Caspase-1 Detection Kit (Immunochemistry, Bloomington, MN, USA). After 48 h exposure to APAP (10 mM), L-02 cells were harvested in 1.5 ml centrifuge tubes and incubated with FAM-YVAD-FMK for 60 min at 37°C in the dark. The cells were then stained with 1 μM SYTOX^®^ Blue stain (Molecular Probes, Eugene, OR, USA) for a further 10 min at room temperature to detect membrane pore formation. After staining, cells were analyzed using a flow cytometer (FC) (BD Biosciences, San Jose, CA, USA), and inflammasomes were defined as double positive for FAM-YVAD-FMK and SYTOX^®^ Blue stain.

### Lactate Dehydrogenase Release

Lactate dehydrogenase (LDH) levels in supernatants were determined using a LDH cytotoxicity detection kit (Roche, Basel, Switzerland) according to manufacturer’s protocols. The absorbance was read at 490 nm on a microplate reader (Tecan).

### Statistical Analysis

SPSS 21.0 software was used for *one-way ANOVA* tests among groups. The data was expressed as mean ± SEM. Statistical differences were represented as P < 0.05, P < 0.01, and P < 0.001.

## Results

### Fisetin Protects Against Acetaminophen-Induced Liver Damage in Mice

APAP induced levels of ALT/AST and MPO, ROS, MDA in mice liver tissues were significantly increased, whereas they decreased significantly in the presence of FST (**Figures 1B–F**) at 6 h. Hematoxylin & eosin staining (**Figure 1C**) showed that APAP led to serious hepatic damage, induced by hepatocyte necrosis, intrahepatic hemorrhage, lymphocyte infiltration, and liver structure destruction, whereas they were ameliorated by FST. FST enhanced GSH levels in mice liver when compared to APAP. This observation further confirmed the protecting effects of FST in contradiction of APAP-induced hepatic damage (**Figure 1G**). In addition, mice treated with FST alone did not show any obvious differences when compared with the vehicle group.

**Figure 2:**
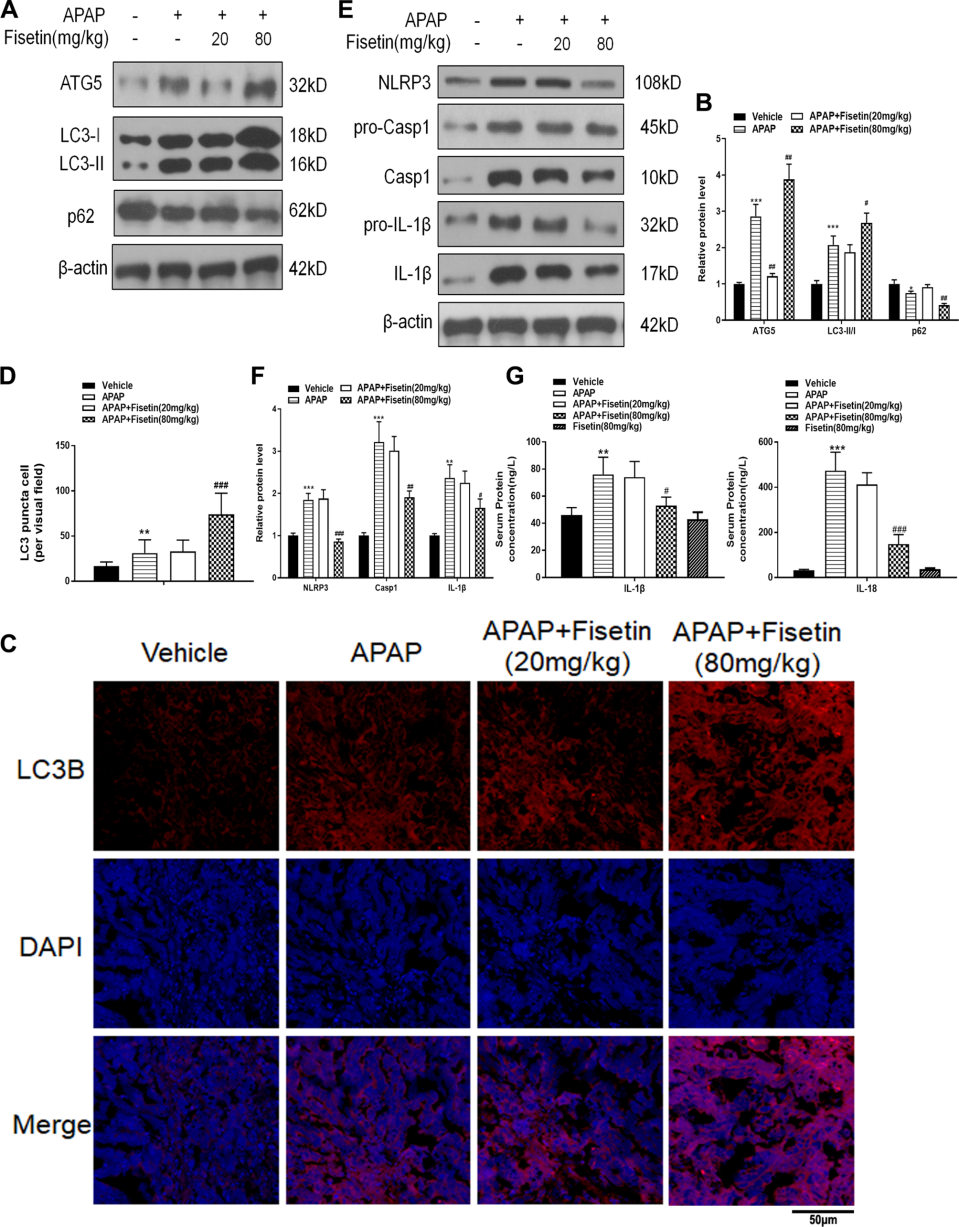
Fisetin (FST) promotes autophagy and inhibits inflammasome activation. **(A)** Western blot of autophagy-related mouse liver proteins. **(B)** Quantification of Western blot. **(C)** A representative image of LC3 puncta (red) staining in liver tissue. Nuclei were counterstained with 4′,6-diamidino-2-phenylindole (DAPI) (blue). **(D)** Quantification of liver tissue LC3 puncta-positive cells. **(E)** Western blot of inflammasome-related proteins. **(F)** Quantification of Western blot. **(G)** Mouse serum interleukin (IL)-1β and IL-18 levels as determined by ELISA. Data are expressed as mean ± SEM (n = 8). **P < 0.01, ***P < 0.001 compared to vehicle; ^##^P < 0.01, ^###^P < 0.001 compared to APAP.

Data from **Figure 2** showed that APAP upregulated the expression of the autophagy related proteins: ATG5 and LC3-II in liver tissues. APAP also downregulated p62 as detected by western blotting, whereas the administration of FST (80 mg/kg) appeared to promote expression of this protein in liver tissues (**Figures 2A, B**). To further characterize autophagosome formation, we examined LC3-containing puncta by immunofluorescence. In the vehicle group, there was slight accumulation of LC3 puncta in mice liver tissue. However, after APAP administration, a large increase in LC3 puncta accumulation was observed, and notably FST at 80 mg/kg aggravated this process as those with autophagy related proteins compared with APAP group (**Figures 2C, D**). APAP alleviated the inflammasome related proteins NLRP3, Casp1, and IL-1β in mice liver tissues, whereas FST (80 mg/kg) reduced these levels as determined by western blotting (**Figures 2E, F**). Using ELISA, IL-1β, and IL-18 concentrations in mice serum were significantly increased in the presence of APAP at 6 h. These levels were reduced by FST (80 mg/kg) (**Figure 2G**). From these observations, it would appear that FST protected against APAP-induced liver damage *in vivo*, as well as promoting autophagy and inhibiting inflammasome related functions.

### Fisetin Prevents Acetaminophen-Induced Cytotoxicity *In Vitro*

**Figure 3:**
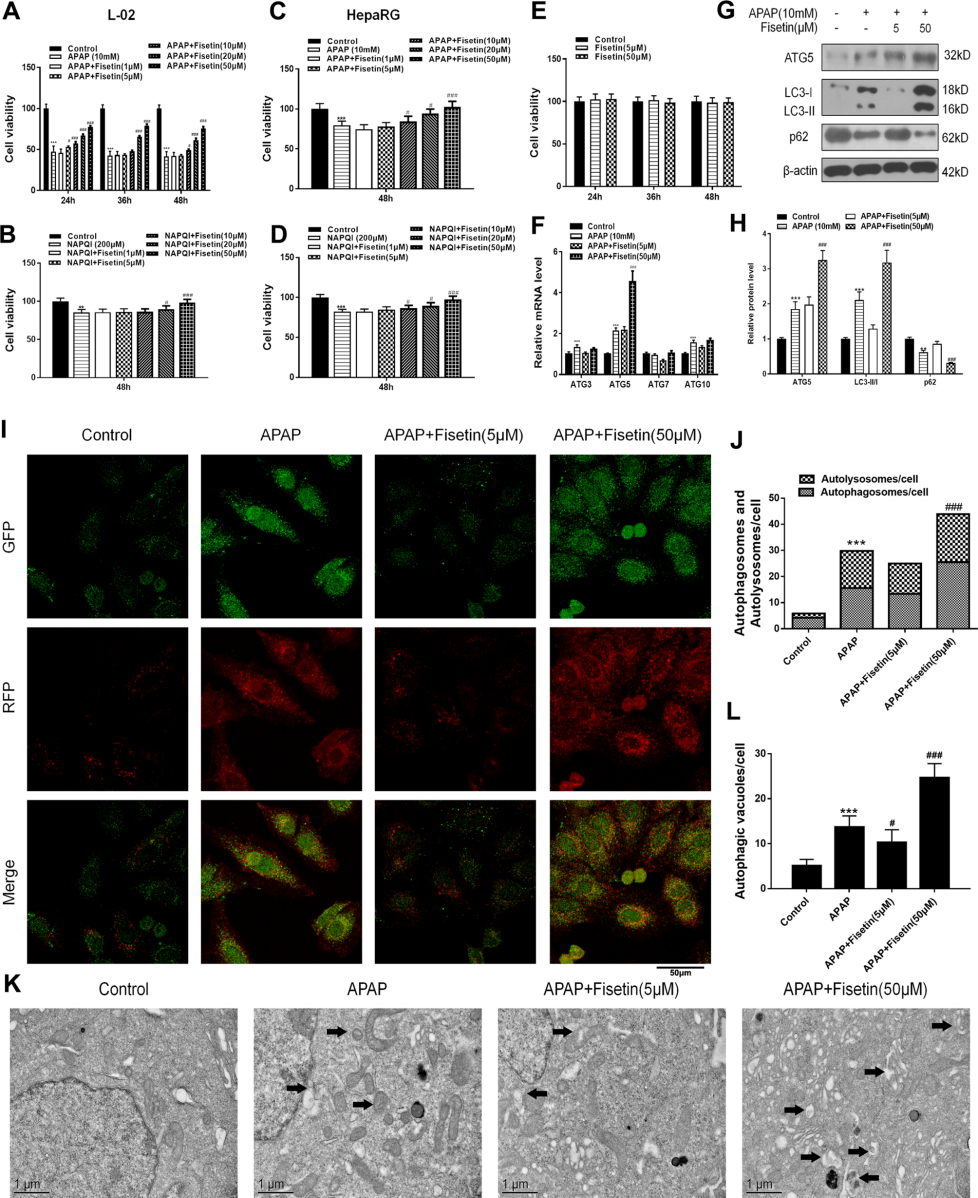
Fisetin (FST) attenuates acetaminophen (APAP)-induced cytotoxicity by promoting autophagy and inhibiting inflammasome activation in L-02 cells. **(A, B)** Cell viability was assessed by MTT assay in response to APAP (10 mM) or N-acetyl-p-benzoquinoneimine (NAPQI) (200 μM) exposure for different periods in L-02 cells. **(C, D)** Cell viability was assessed by MTT assay in response to APAP (10 mM) or NAPQI (200 μM) exposure for 48 h in HepaRG cells. **(E)** Cell viability was assessed by MTT assay for different concentration of FST in L-02 cells. **(F)** Messenger RNA (mRNA) expression of ATG genes was determined by RT-PCR in the presence of APAP (10 mM) for 48 h. **(G)** Western blot of autophagy-related proteins in L-02 cells supplemented with APAP (10 mM) for 48 h. **(H)** Quantification of Western blot. **(I, J)** Representative immunofluorescence images of mRFP-GFP-LC3 in L-02 cells supplemented with APAP (10 mM) for 48 h. Representative profiles of autophagosomes (RFP+GFP+dots) and autolysosomes (RFP+GFP-dots) per cell section, assessed by confocal microscopy are shown and were quantified. **(K, L)** Autophagic vacuoles (autophagosomes) were assessed by transmission electron microscopy (TEM) plus APAP (10 mM) for 48 h in L-02 cells. Representative TEM images are shown and typical autophagosomes are marked with black arrows. Autophagosome numbers per cell were calculated by counting the number of double-membrane organelles in 10 cells. Data are expressed as mean ± SEM (n = 3). **P < 0.01, ***P < 0.001 compared to control; # < 0.05, ### < 0.001 compared to APAP.

As shown in **Figures 3A–D**, APAP (10 mM) and the metabolic product of APAP, NAPQI (200 µM) both decreased cell viability of L-02 and HepaRG cells, when compared to control cells. HepaRG is a preferable cell model for drug-induced liver injury (DILI) (Wu et al., 2016). However, FST reversed the decreased cell viability induced by APAP and NAPQI in L-02 and HepaRG cells in a concentration-dependent manner. Besides, we detect cell viability in L-02 cells only treated with FST. The results indicated no loss of viability was observed in both FST 5 and 50 μM. Next, we detected messenger RNA (mRNA) levels of several autophagy related proteins using RT-PCR in L-02 cells. Although APAP (10 mM) enhanced ATG3, ATG5, and ATG10 expression, only ATG5 expression was dramatically increased by FST (50 μM) (**Figure 3F**). Next, we confirmed this observation using western blotting, and found that ATG5 expression was enhanced in the presence of FST (50 μM), consistent with other observations in this study. We then examined ATG5 downstream pathways; western blotting showed that FST (50 μM) enhanced APAP-induced increases in LC3-II, downregulated p62, suggesting that FST promoted APAP-induced autophagy (**Figures 3G, H**).

To determine autophagic effects in L-02 cells, mRFP-GFP-LC3, a specific marker for autophagosomes and autolysosomes, was transfected into cells treated with APAP and FST for 48 h. As expected, we found that cells treated with APAP showed typically dense accumulations of mRFP-LC3 and GFP-LC3 puncta. These mRFP-GFP-LC3 puncta dramatically increased upon APAP exposure. FST (50 μM) enhanced this accumulation when compared with the APAP group (**Figures 3I, J**). Transmission electron microscopy (TEM) of autophagic vacuoles from L-02 cells confirmed these results (**Figures 3K, L**).

**Figure 4:**
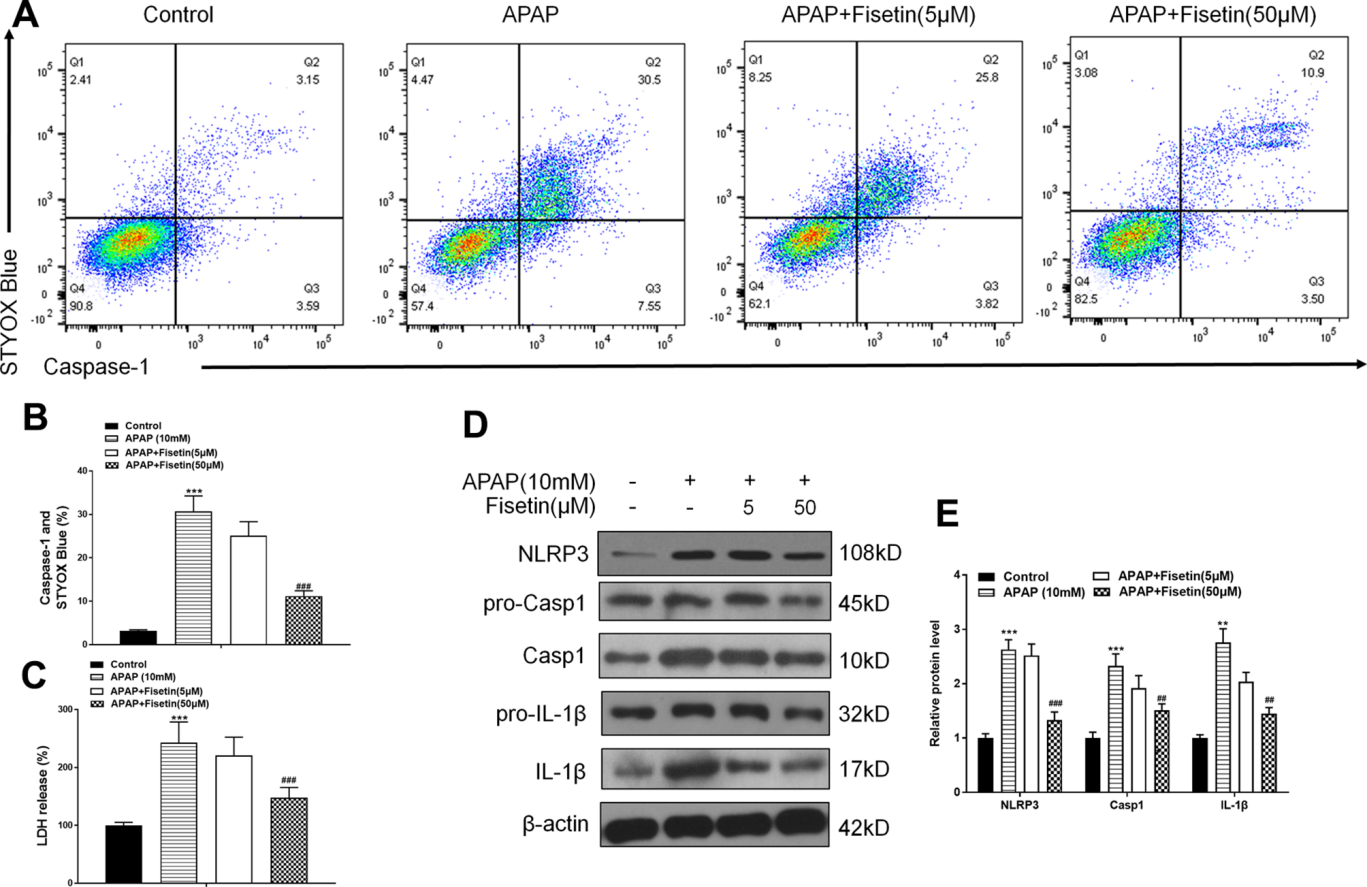
Fisetin (FST) inhibits inflammasome activation in L-02 cells. **(A, B)** Analysis and quantification of caspase-1 activation rates by FC with acetaminophen (APAP) (10 mM) for 48 h in L-02 cells. **(C)** Lactate dehydrogenase (LDH) release plus APAP (10 mM) for 48 h in L-02 cells. **(D)** Western blot of inflammasome-related proteins plus APAP (10 mM) for 48 h in L-02 cells. **(E)** Quantification of Western blot. Data are expressed as mean ± SEM (n = 3). ^***^P < 0.001 compared to control; ^###^P < 0.001 compared to APAP. Data are expressed as mean ± SEM (n = 3). ^**^P < 0.01, ^***^P < 0.001 compared to control; ^#^P < 0.05, ^###^P < 0.001 compared to APAP.

Recent evidence has suggested that the means of validating inflammasomes is detecting caspase-1 activation and lactate dehydrogenase (LDH) release (Wree et al., 2014). FC was used to detect caspase-1 activation and dead cell formation in hepatic cells. The FC results demonstrated that L-02 cells, treated with APAP, showed a marked increase in the number of caspase-1 and SYTOX Blue (indicating dead cell) double-positive cells (**Figures 4A, B**). APAP exposure also resulted in increased LDH release from L-02 cells, which was suppressed by FST (50 μM) (**Figure 4C**). Western blotting showed that FST (50 μM) downregulated increases, induced by APAP, in inflammasome related proteins, NLRP3, Casp1, and IL-1β (**Figures 4D, E**).

These data suggested that FST prevented APAP-induced cytotoxicity *in vitro,* promoted autophagy, and downregulated inflammasome-related protein expression.

### Fisetin Prevents Cytotoxicity Through ATG5 in L-02 Cells

**Figure 5:**
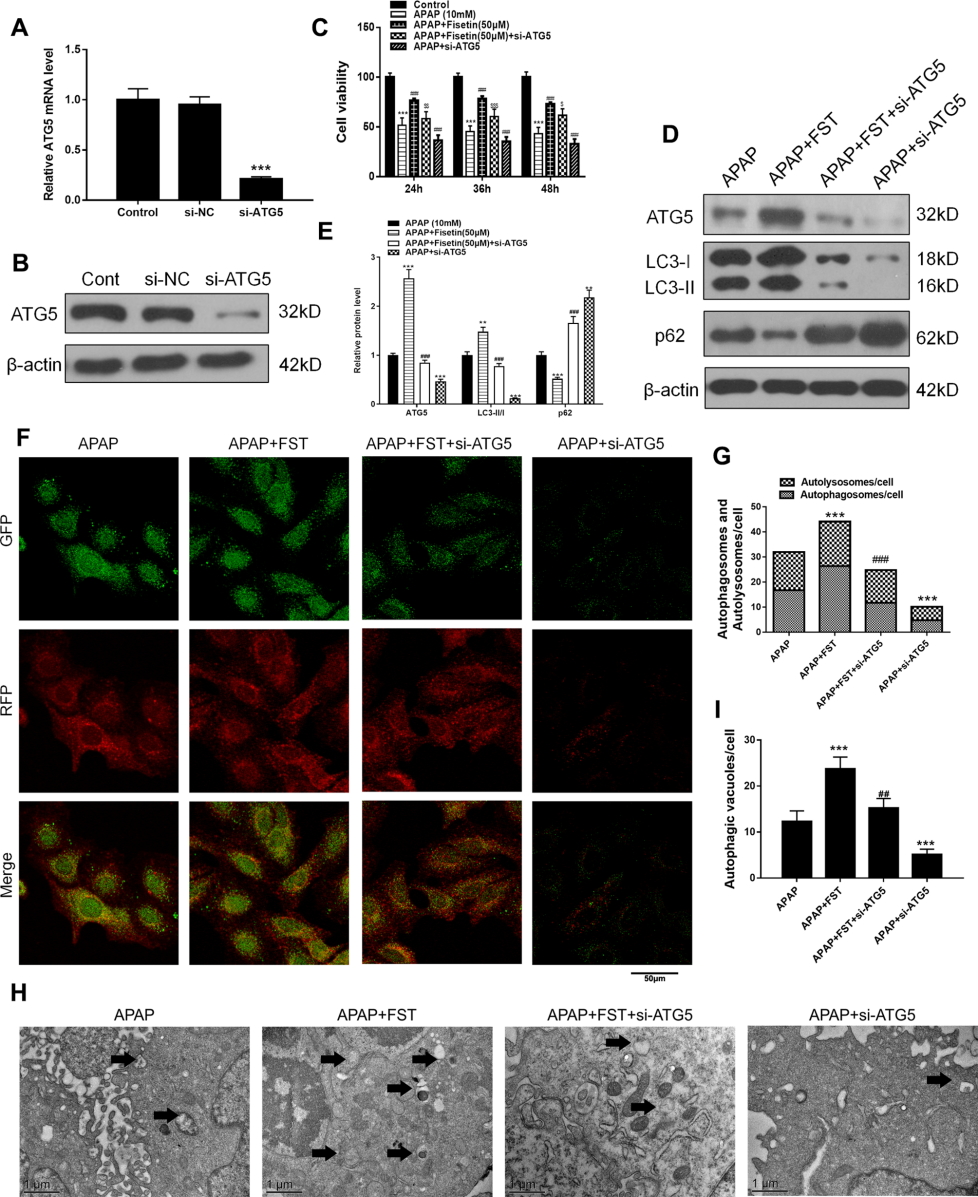
Fisetin (FST) attenuates acetaminophen (APAP)-induced cytotoxicity *via* ATG5 in L-02 cells. **(A, B)** ATG5 messenger RNA (mRNA) expression was determined by real time (RT)-PCR and western blotting. **(C)** Cell viability was assessed by MTT assay in response to APAP exposure for different times in L-02 cells. **(D)** Western blot of autophagy-related proteins in cells plus APAP (10 mM) for 48 h. **(E)** Quantification of Western blot. **(F, G)** Representative immunofluorescence images of mRFP-GFP-LC3 in cells plus APAP (10 mM) for 48 h. Representative profiles of autophagosomes (RFP+GFP+ dots) and autolysosomes (RFP+GFP-dots) per cell section, as assessed by confocal microscopy are shown and were quantified. **(H, I)** Autophagic vacuoles (autophagosomes) were assessed by transmission electron microscopy (TEM) plus APAP (10 mM) for 48 h. Representative TEM images are shown and typical autophagosomes are marked with black arrows. Autophagosome numbers per cell were calculated by counting double-membrane organelles in 10 cells. Data are expressed as mean ± SEM (n = 3). **P < 0.01, ***P < 0.001 compared to control or APAP; ^#^P < 0.05, ^###^P < 0.001 compared to APAP or AAP+FST; ^$^P < 0.05, ^$$^P < 0.01, ^$$$^P < 0.001 compared to APAP+FST.

To investigate whether FST plays a role through the ATG5 pathway, we constructed si-ATG5 to inhibit ATG5 expression. As expected, both RT-PCR and western blotting data showed that si-ATG5 significantly downregulated ATG5 expression (**Figures 5A, B**).

FST increased the numbers of surviving hepatocytes after incubation with APAP for 48 h, whereas si-ATG5 lowered these numbers (**Figure 5C**). Western blotting revealed that for proteins in the ATG5 downstream pathway, si-ATG5 reversed FST effects by downregulating LC3-II, while upregulating p62 (**Figures 5D, E**). mRFP-GFP-LC3 and TEM observations also showed that si-ATG5 decreased autophagosomes and autolysosomes in L-02 cells and reversed the protective effects of FST against APAP (**Figures 5F–I**).

**Figure 6:**
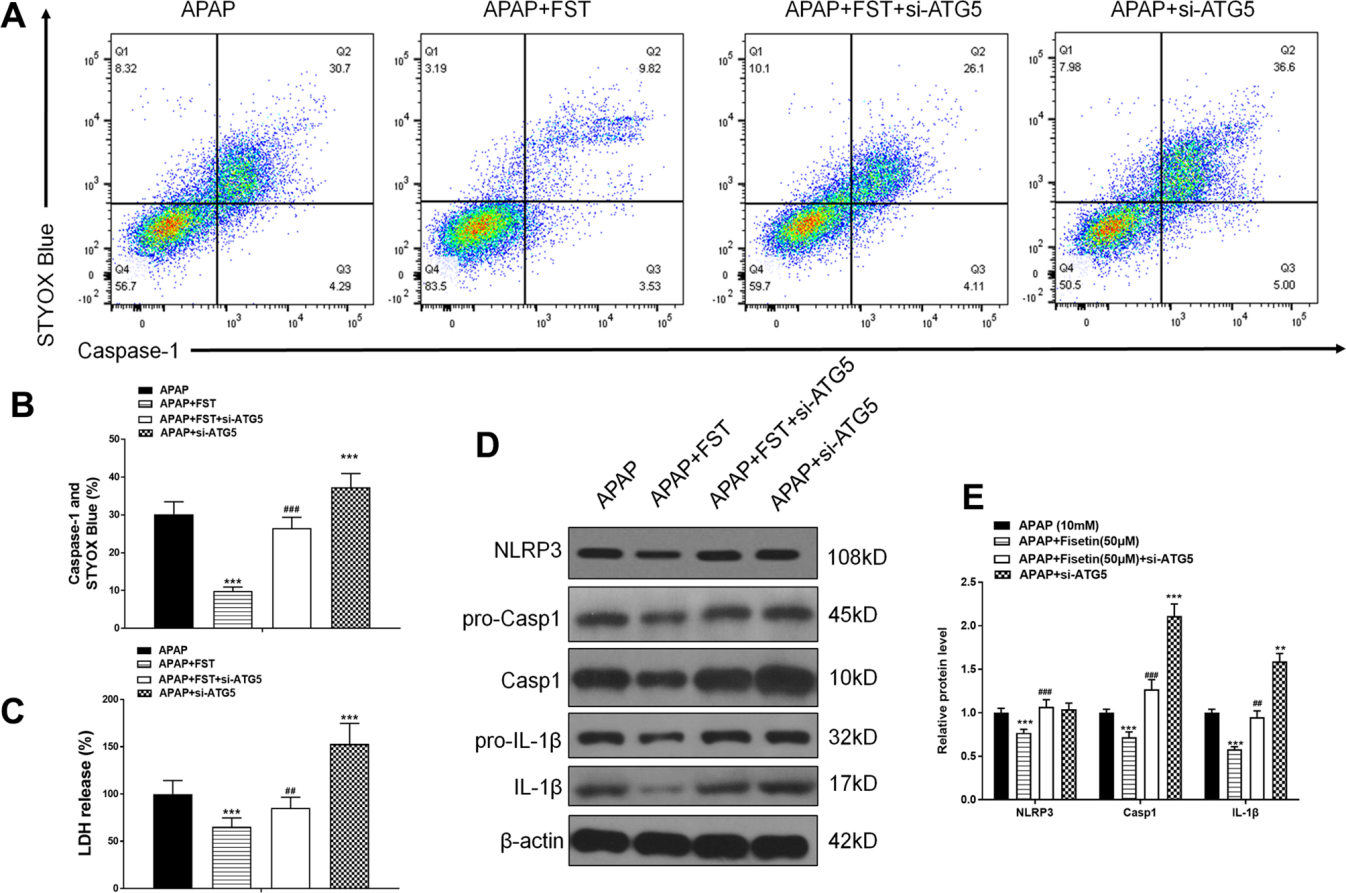
Fisetin (FST) inhibits inflammasome activation *via* ATG5 in L-02 cells. **(A, B)** Analysis of caspase-1 activation rates by flow cytometry with acetaminophen (APAP) for 48 h and quantified. **(C)** Lactate dehydrogenase (LDH) release with APAP for 48 h. **(D)** Western blot of inflammasome-related proteins with APAP for 48 h. **(E)** Quantification of Western blot. Data are expressed as mean ± SEM (n = 3). ***P < 0.001 compared to control or APAP; ## < 0.01, ### < 0.001 compared to APAP or AAP+FST.

Our FC results showed that si-ATG5 increased the decreased double positivity for active caspase-1 and STYOX blue caused by FST (**Figures 6A, B**). Exposure to si-ATG5 led to increased LDH release from L-02 cells when compared to cells incubated with FST alone (**Figure 6C**). Similarly, western blotting demonstrated that si-ATG5 upregulated FST-mediated decreases in inflammasomes proteins NLRP3, Casp1, and IL-1β (**Figures 6D, E**). In addition, cells treated with si-ATG5 alone aggravated damage induced by APAP. Accordingly, these data suggested that FST played a role *via* the ATG5 pathway.

### Fisetin Inhibits Activation of Inflammasomes by Promoting Autophagy in L-02 Cells

**Figure 7:**
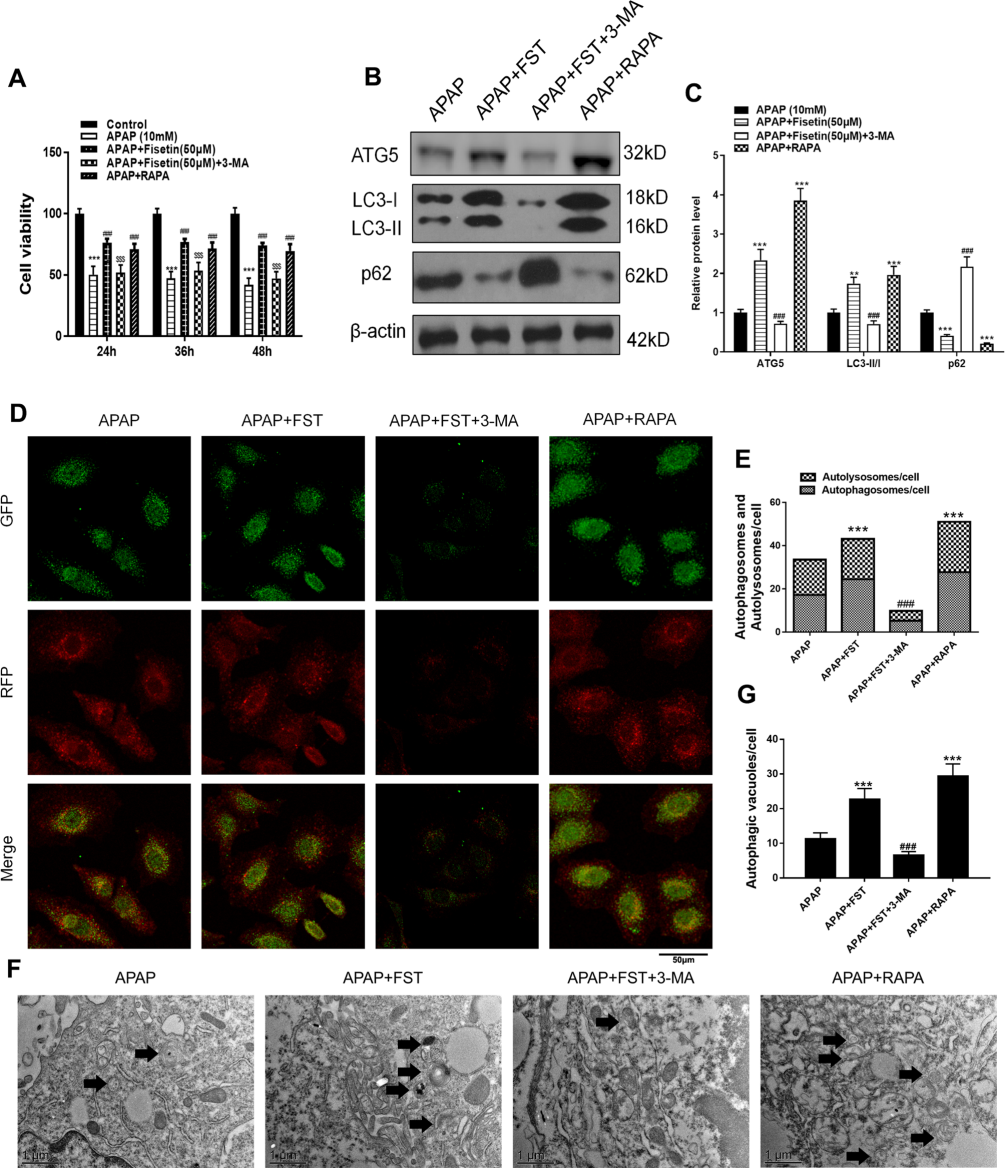
Inhibiting autophagy reverses the protective effects of fisetin (FST) in L-02 cells. **(A)** Cell viability was assessed by MTT assay in response to acetaminophen (APAP) (10 mM) exposure for different times in L-02 cells, pretreated with 3-methyladenine (3-MA) (5 mM) or rapamycin (RAPA) (100 nM) for 1 h. **(B)** Western blot of autophagy-related proteins in L-02 cells plus APAP (10 mM) for 48 h. **(C)** Quantification of Western blot. **(D, E)** Representative immunofluorescence images of mRFP-GFP-LC3 in L-02 cells plus APAP (10 mM) for 48 h. Representative profiles of autophagosomes (RFP+GFP+ dots) and autolysosomes (RFP+GFP-dots) per cell section, as assessed by confocal microscopy are shown and were quantified. **(F, G)** Autophagy vacuoles (autophagosomes) were detected by transmission electron microscopy (TEM) plus APAP (10 mM) for 48 h. Representative TEM images are shown and typical autophagosomes are marked with black arrows. Autophagosome numbers per cell were calculated by counting double-membrane organelles in 10 cells. Data are expressed as mean ± SEM (n = 3). **P < 0.01, ***P < 0.001 compared to control or APAP; ^#^P < 0.05, ^###^P < 0.001 compared to APAP or APAP+FST; ^$^P < 0.05, ^$$^P < 0.01, ^$$$^P < 0.001 compared to APAP+FST.

Results revealed that FST reversed reductions in APAP-mediated cell viability, while treatment of cells with the autophagy promoter, rapamycin (RAPA), revealed similar cell viabilities to FST treatments. However, 3-methyladenine (3-MA) lowered cell viability, thus proving that autophagy could increase cell viability (**Figure 7A**). Western blotting demonstrated that 3-MA inhibited FST-induced autophagy, while RAPA further promoted autophagy when compared with APAP (**Figures 7B, C**).

mRFP-GFP-LC3 and TEM observations also showed that 3-MA decreased autophagosomes and autolysosomes in L-02 cells, indicating that the autophagy inhibiter inhibited FST-induced autophagy, whereas RAPA promoted autophagy (**Figures 7D–G**).

**Figure 8:**
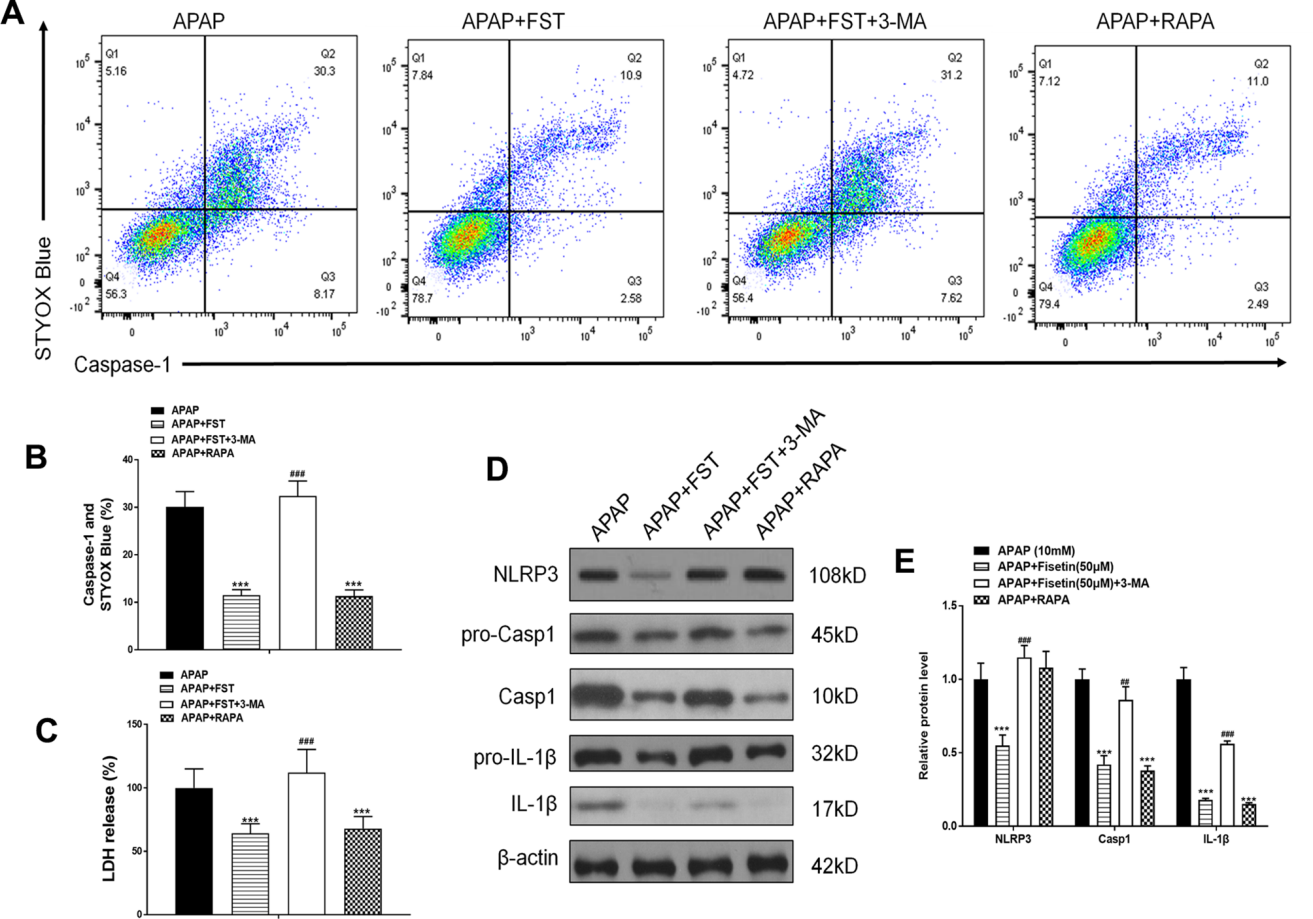
Inhibiting autophagy reverses the inhibiting effect of fisetin (FST) on inflammasomes, in L-02 cells. **(A, B)** Analysis of caspase-1 activation rates by flow cytometry with acetaminophen (APAP) for 48 h and quantified. **(C)** Lactate dehydrogenase (LDH) release with APAP for 48 h. **(D)** Western blot of inflammasome-related proteins with APAP for 48 h. **(E)** Quantification of Western blot. Data are expressed as means ± SEM (n = 3). ***P < 0.001 compared to control or APAP; ## < 0.01, ### < 0.001 compared to APAP or AAP+FST.

To investigate the relationship between autophagy and inflammasomes, we used RAPA and 3-MA. As FC results showed, 3-MA increased the decreased double positivity for active caspase-1 and STYOX blue caused by FST, whereas RAPA lowered these levels (**Figures 8A, B**). 3-MA increased LDH release while RAPA appeared to downregulate LDH levels (**Figure 8C**). Likewise, 3-MA increased the expression of inflammasome proteins NLRP3, Casp1, and IL-1β, and *vice versa* (**Figures 8D, E**).

From these observations, we suggest that FST inhibits the activation of inflammasomes and protects APAP-induced hepatocyte damage by promoting autophagy.

## Discussion

FST is a flavonoid polyphenol found in plants and vegetables. It is believed to have anti-tumor, anti-oxidation, anti-inflammatory, and other pharmacological effects. In our study, we demonstrated that FST protected hepatic injury caused by acetaminophen *in vivo* and *vitro* by inducing autophagy *via* the ATG5 pathway.

Autophagy is a highly conserved mechanism in cellular homeostasis. The process consists of five distinct stages: initiation, elongation, autophagosome formation, and autophagosome fusion with lysosomes, followed by degradation (Yang and Klionsky, 2009). These steps are regulated by autophagy-related genes (ATG). Up to the present time, more than 30 ATG family members have been acknowledged, and their functions have been studied in- depth. ATG5 is an important gene involved in autophagy formation; it is located on human chromosome 6q21, is 276 amino acids long and contains approximately 384 nucleotide polymorphisms (Jounai et al., 2007).

Autophagosome formation incorporates 16 autophagy-related (Atg) genes, including Atg5, which comprises an ubiquitin-like conjugation system (Mizushima et al., 2011). The conversion of a cytosolic truncated form of LC3 (LC3-I) to its autophagosomal membrane-associated, phosphatidylethanolamine-conjugated form (LC3-II), visible as discrete puncta by immunofluorescent analysis, indicates autophagosome formation (Ravikumar et al., 2010). The protein lipidation system, resulting in LC3-II, is driven by the Atg5-Atg12-Atg16 complex which acts as an E3 ligase equivalent that facilitates the localized conversion of LC3-I into LC3-II (Yang et al., 2010). In this study, we found APAP stimulated the ATG family (**Figure 3E**). Several studies, within the context of APAP-induced autophagy, have indicated the possibility that APAP may inhibit mTOR activity and decrease cellular ATP levels to trigger AMPK activation (Ni et al., 2013; Mo et al., 2018). Therefore, we speculate that APAP activates the AMPK pathway and regulate the ATG family (Bordi et al., 2019; Xing et al., 2019).

We also demonstrate that FST promotes autophagy *via* the ATG5 pathway, through the inhibition of ATG5 expression (si-ATG5). We note that autophagy activity was significantly reduced when compared to controls. A previous study has shown that FST promoted autophagic degradation of phosphorylated tau *via* the activation of TFEB and Nrf2 transcription factors (Kim et al., 2016), suggesting that FST exerts pro-autophagy effects. However, another study has also shown that FST exerts autophagy repressing effects (Yen et al., 2017) at doses < 20 µM in tunicamycin-mediated cells. This observation appears contrary to our conclusions, yet consistent with data from **Figures 3** and **4**, where we show that FST (5 μM) decreased L-02 cell viability, inhibited autophagy and upregulated inflammasome associated proteins. This phenomenon suggests that different FST doses can lead to different pharmacological effects. Here, 5 μM FST inhibited autophagy while 50 μM did the opposite. We have demonstrated that different FST levels can promote autophagy, which improves damage caused by APAP in hepatocytes, as observed in previous work (Hu et al., 2018; Mo et al., 2018; Shan et al., 2018).

Autophagy and inflammation are innate immune pathways that regulate each other and protect the body from invading pathogens. In addition to controlling the metabolic balance of cells through nutrient circulation, the “self-cannibalization” process of autophagy is also responsible for degrading damaged organelles, aggregated protein complexes and pathogens, all to protect intracellular integrity. Recently, it was reported that autophagy regulates the activation of inflammatory corpuscles (Saitoh et al., 2008), where Atg16L1 deletion significantly increased the activation of inflammasomes in LPS-induced macrophages. Subsequently, it was reported that 3-MA increased the activation of NLRP3 inflammasomes in THP-1 macrophages (Zhao et al., 2011). In addition to 3-MA, ATG7, or ATG5 silencing led to inflammasome activation, further suggesting autophagy regulation mechanisms for the activation of inflammasomes. According to a previous study (Williams-Bey et al., 2014), the activation of inflammasomes in macrophages exposed to DHA is hampered, while ATG7 defective (silenced) cells are resistant to DHA inhibition. Although it is generally believed that autophagy can regulate the activation of inflammasomes, the mechanism is still unknown; however, studies have shown that ROS accumulation, mitochondrial DNA, and inflammasome degradation processes are involved.

Many studies have shown that inflammasomes plays vital roles in APAP-induced liver injury (Williams et al., 2011; Woolbright and Jaeschke, 2017; Kim and Park, 2018). When inflammasomes are activated, the primary outcome is caspase-1 expression and the cleavage of pro-IL-1ß to IL-1ß, which binds to IL-1R thereby stimulating the hepatic recruitment of IL-1R expressing cells, resulting in inflammation (Woolbright and Jaeschke, 2017). This leads to intrahepatic hemorrhage, lymphocyte infiltration, and the destruction of liver structures. Therefore, the inhibition of inflammasomes appears to be beneficial for APAP-induced liver damage. Our research suggests that promoting autophagy can lead to the inhibition of inflammasomes. The autophagy promoter, RAPA inhibited inflammasomes and increased cell viability, whereas the autophagy inhibitor 3-MA did the opposite (**Figures 7** and **8**). A similar study showed that the autophagy inducer carvedilol inhibited NLRP3 inflammasomes (Wong et al., 2018), which is consistent with our results. Recent evidence suggests that active inflammasomes also promote cell pyroptosis, which is caspase-1 dependent and differs from other types of cell death. However, pyroptosis has been largely unexplored in liver injury, especially APAP-induced liver injury. Therefore, we in future work we will deeply investigate FST effects in cell pyroptosis in APAP hepatotoxicity.

## Conclusions

We have shown that FST promotes autophagy through the ATG5 pathway, thus inhibiting inflammasomes by promoting autophagy, potentially reversing liver-mediated APAP injury.

## Data Availability Statement

The datasets generated for this study are available on request to the corresponding authors.

## Ethics Statement

All animals received humane care in compliance with institutional animal care guidelines approved by the Experimental Animal Ethical Committee of Shanghai University of Traditional Chinese Medicine. The protocol was reviewed and approved by the Experimental Animal Ethical Committee of Shanghai University of Traditional Chinese Medicine (Permit Number: PZSHUTCM190315014).

## Author Contributions

JZ, LZ, and CH: performed the research. GJ, QC, and YJ: designed the research study. TW, JL, and CW: contributed essential reagents or tools. LC, MJ, and HH: collected the data. JZ and CH: analyzed the data. LZ and JZ: wrote the paper.

## Funding

This work was financially supported by the National Natural Science Foundation of China (81703879); Shanghai Municipal Commission of Health and Family Planning general project for clinical research of health industry (201840377, 201940449); Putuo District of Shanghai Science And Technology Commission Research Project (ptkwws201813); the Budget Project of Shanghai University of Traditional Chinese Medicine (2016YSN60); the Budget of Experiment Center for Science and Technology (18LK022).

## Conflict of Interest

The authors declare that the research was conducted in the absence of any commercial or financial relationships that could be construed as a potential conflict of interest.
